# Influence of Interferon beta treatment on quality of life in multiple sclerosis patients

**DOI:** 10.1186/1477-7525-4-96

**Published:** 2006-12-12

**Authors:** Isabella Laura Simone, Antonia Ceccarelli, Carla Tortorella, Alessandra Bellacosa, Fabio Pellegrini, Immacolata Plasmati, Maria Fara De Caro, Mariangela Lopez, Francesco Girolamo, Paolo Livrea

**Affiliations:** 1 Department of Neurological and Psychiatric Sciences, University of Bari, Italy; 2 Department of Clinical Pharmacology and Epidemiology, Istituto di Ricerche Farmacologiche Mario Negri, Consorzio Mario Negri Sud, Santa Maria Imbaro, Italy

## Abstract

**Background:**

Interferon-beta (IFN-β) shows beneficial effect on the course of multiple sclerosis (MS), nevertheless its route and frequency of administration and side effects might impact negatively the quality of life (QoL) of MS patients. The objective of this study was to evaluate the influence of IFN-β on QoL in MS patients.

**Methods:**

Seventy-seven disease modifying treatment (DMT) free and 41 IFN-β treated MS patients were evaluated. QoL, assessed by MSQoL-54, was related to IFN-β treatment and to clinical and demographic parameters at baseline and after two years. Multivariate hierarchical linear model for repeated measurements was used.

**Results:**

Treated patients showed a younger age, a lower disease duration and a higher relapse rate in the two years preceding study entry. At inclusion time treated and untreated patients did not differ in relapse rate, expanded disability status scale (EDSS), fatigue, depression, physical and mental QoL. IFN-β did not influence QoL at inclusion time, but when QoL was evaluated after two years, treatment negatively affected mental QoL. Depression and fatigue negatively influenced physical and mental QoL both at baseline and after two years. EDSS correlated with a poor physical QoL only at baseline.

**Conclusion:**

IFN-β had a negative impact on QoL over the time in MS patients, influencing mainly mental QoL. The impairment of QoL in MS was strongly associated with increasing fatigue and depression, whereas clinical disability had a minor unfavourable role.

## Background

In the last years increasing interest focused on QoL instrument as a broader measure of the burden of MS. Recent studies reported that QoL assessment may provide unique information often underestimated by the EDSS [1-3], which remains to date the primary clinical outcome measure for MS related disability [4]. Several factors may influence the QoL in MS patients. Previous studies showed varying results on the relationship between neurological disability and impaired QoL, suggesting only a partial contribute of disability on QoL. On the contrary fatigue and depression are highly associated to a poor QoL [5-11].

Although the QoL instrument is recommended as an outcome measure of therapeutic choices and treatment effectiveness, its use is still limited in the routine clinical approaches [12]. Patient's perspectives derived from lifelong treatment, as well as adherence to prolonged therapies and side-related effects are often underestimated. Some authors reported a beneficial influence of IFN-β therapy on QoL [13,14]. Nevertheless, adverse effects related to treatment may negatively impact QoL [14,15].

The objective of this study was to determine the influence over the time of IFN-β treatment on QoL in MS patients.

## Methods

### Patients

A non-randomized study was performed on 127 consecutive patients followed at MS centre of the Department of Neurological and Psychiatric Sciences of the University of Bari. The patients were enrolled over three months. At inclusion time, QoL was evaluated in all patients. After two years further assessment of QoL was proposed to the same patients: 93 out of 127 completed the MS QoL questionnaire, whereas 34 patients refused. Seventy-seven of 127 patients were DMT free and not eligible for immunosuppressive or immunomodulatory therapies during the following two years. Forty-one patients were assuming IFN-β from 3.6 ± 1.9 months at inclusion: 30 patients IFN-β 1a (14 Avonex^® ^and 16 Rebif ^®^) and 11 patients IFN-β 1b. No patient stopped treatment during two years of follow-up. Finally 9 of 127 patients started to assume IFN-β at different time during the study. This latter group was excluded from the analysis, since the relative low number of patients and the limited use of the drug might be confounding factors for statistical evaluation.

All patients had definite MS, according to Mc Donald criteria [16]. Exclusion criteria were current or past history of psychiatric disorders, clinical relapse or corticosteroids drugs in the last three months preceding the observation. No patient needed anti-depressive treatment for the whole period of the study. The study was conducted with local ethical committee approval and with patients written informed consent.

### Measures

In all patients clinical disability was measured by the EDSS [4]. Quality of life was assessed by MSQoL-54 instrument developed by Vickrey et al [17] and validated in an Italian population by Solari at al [18]. The scale consists of 54 items that are distributed in 12 multi-item scales and 2 single item scales. The instrument includes questions from Short Form 36-Item Health Survey as a generic core measure [19] and 18 additional items specific for MS exploring health distress, sexual function, overall quality of life, cognitive function and energy. Physical and mental health composite scores are calculated as a weighted sum of selected domains to generate a simplified two-dimension solution to MSQoL-54 instrument.

The subscales for the physical health composite summary are: physical function, health perceptions, energy, role limitation-physical, bodily pain, social function and health distress. The subscales for the mental health composite summary are: overall quality of life, emotional well being, role limitation-emotional, cognitive function and health distress. The composite scores range from 0 (poor health) to 100 (optimal health).

Fatigue severity scale (FSS) consists of nine questions, ranging from 1 (low fatigue) to 7 (high fatigue) with a suggested cut-off point of 4.5 [20]. Depression status was assessed using the Beck Depression Inventory (BDI) consisting of 21 items self-report rating inventory [21]. Cognitive functions were evaluated by Mini Mental State Examination corrected with school-attendance index (MMSE-c) [22].

MSQoL-54, FSS and BDI were self-administered in the presence of an assistant if required by the patients.

### Statistical analysis

Patients' baseline characteristics in treated and untreated groups were compared using Pearson χ^2 ^for categorical variables and t-student test for continuous variables.

Baseline and changes after two years in clinical items and QoL scores were expressed as mean ± standard deviation (SD). T-student test was used to assess differences between treated and untreated groups at baseline and after two years of follow-up for all continuous measures. Each MSQoL-54 component (physical and mental composite score and relative scales) was analysed with a multivariate hierarchical linear model for repeated measurements [23]. Hierarchical linear models are particularly suited for repeated measurements analysis dealing with unbalanced designs (i.e. presence of drop-outs). These models allow to simultaneously assess the impact of baseline and time-varying covariates on QoL baseline scores and their changes over time, and also to evaluate, via interaction terms with the time variable, the impact of baseline covariates over time.

For each QoL component the baseline covariates were: age at inclusion, gender (male vs. female), disease duration, disease course (secondary progressive, SP vs. relapsing remitting, RR), relapse rate in the two years preceding the study entry, EDSS, BDI, FSS, MMSE-c and IFN-β treatment (IFN-β treated vs. untreated groups). Changes in time-varying covariates are expressed as difference between two years follow-up and baseline assessment. The impact of changes in relapse rate, EDSS, BDI, FSS and MMSE-c on changes in QoL scores and the effect of treatment group over the time were also evaluated.

As to baseline variables, β parameters for continuous covariates represent the difference in QoL score for each unit increase in the covariate, and βs for categorical covariates indicate the difference in QoL for each category respect to the reference one. As to other variables, βs for time-varying covariates refer to QoL changes for each unit change in the covariate over the two years of follow-up, and βs for time and treatment-by-time interaction indicate QoL changes over the two years of follow-up in treated and untreated groups. P-values < 0.05 were considered significant.

Further, drop-out mechanism was analysed to assess the extent of informative missing data due to patients who did not complete the questionnaire after two years of follow-up [24]. All the analyses were performed using SAS Statistical Package Release 9.1 (SAS Institute, Cary, NC).

## Results

Table 1 reports baseline demographic and disease related patient characteristics according to treatment group. A prevalence of RR course and female gender characterized each group, without any statistical difference. Untreated patients showed a significant higher age at inclusion (p = 0.02) and a longer disease duration (p = 0.04) in comparison to treated patients. At baseline MS groups did not differ in EDSS, BDI, FSS, physical, mental health composite scores as well as relative scales. Relapse rate in the two years before study entry was significantly higher in IFN-β treated patients (p < 0.0001) (Table 2). After two years of follow-up mental health composite score (p = 0.006) and emotional well being score (p = 0.014) significantly decreased in IFN-β treated group (Table 2).

**Table 1:** Baseline characteristics in untreated and treated MS groups

**Variable**	**Covariate**	**Untreated**	**IFN-β treated**	**p-value**
No. of patients		77	41	
Age at disease onset (yrs)		30.3 ± 9.7	28.7 ± 10.5	0.42
Age at inclusion (yrs)		42.1 ± 11.2	36.8 ± 11.5	**0.02**
Gender	Male	28 (36.4)	8 (19.5)	0.06
	Female	49 (63.6)	33 (80.5)	
Disease duration (yrs)		12.0 ± 7.8	8.9 ± 8.1	**0.04**
Disease course	RR	58 (75.3)	34 (82.9)	0.34
	SP	19 (24.7)	7 (17.1)	
School education (degree-yrs)	<5	10 (12.9)	6 (14.6)	0.97
	6–8	16 (21)	7 (17.1)	
	9–13	38 (49.2)	19 (46.3)	
	>13	13 (16.9)	9 (22)	

Data are expressed as means ± SD or n (%). P-values refer to T test or Pearson χ^2^.

**Table 2:** Time-varying covariates. Means ± SD at baseline and change after 2-years follow-up in untreated and treated MS groups

	**Untreated n. 77**		**IFN-β treated n.41**		p-value*	
***Time-varying covariates***	*Baseline*	*Change*	*Baseline*	*Change*	*Baseline*	*Change*
Relapse rate**	0.2 ± 0.3	0.0 ± 0.4	0.7 ± 0.5	-0.1 ± 0.6	**<0.0001**	0.37
EDSS	3.1 ± 1.7	0.4 ± 0.8	3.0 ± 1.8	0.4 ± 1.1	0.84	0.93
BDI	9.5 ± 7.7	1.3 ± 10.3	10.8 ± 9.2	2.2 ± 9.8	0.57	0.88
FSS	2.5 ± 1.8	1.1 ± 1.7	2.8 ± 1.9	1.2 ± 1.8	0.53	0.75
MMSE-c	28.5 ± 2.0	-0.6 ± 2.4	28.1 ± 3.7	-0.5 ± 2.5	0.78	0.70
*MSQoL-54 Measures*						
Physical health composite score	65.5 ± 21.4	-5.5 ± 17.2	65.1 ± 22.3	-9.1 ± 18.9	0.91	0.45
Mental health composite score	69.1 ± 19.8	1.2 ± 20.2	71.6 ± 18.2	-12.1 ± 19.1	0.54	**0.006**
Physical function	67.7 ± 35.8	-2.3 ± 14.7	69.5 ± 35.4	-8.8 ± 19.1	0.80	0.13
Health perceptions	47.5 ± 23.4	-3.0 ± 23.1	48.7 ± 22.7	-6.0 ± 26.9	0.76	0.64
Energy	47.7 ± 22.6	1.1 ± 23.0	47.0 ± 23.0	-1.2 ± 23.0	0.91	0.57
Role limitation-physical	67.6 ± 43.5	-4.6 ± 42.9	65.2 ± 40.7	-14.3 ± 41.8	0.48	0.52
Bodily pain	79.7 ± 25.7	-8.7 ± 30.9	74.0 ± 29.7	-8.1 ± 28.6	0.39	0.41
Sexual function	83.1 ± 26.6	-6.0 ± 35.6	81.5 ± 27.0	-12.0 ± 36.5	0.67	0.62
Social function	74.2 ± 23.3	-5.8 ± 23.4	74.4 ± 21.3	-10.0 ± 21.5	0.87	0.49
Health distress	70.8 ± 23.0	-2.0 ± 31.6	71.9 ± 20.4	-13.3 ± 24.0	0.98	0.06
Overall quality of life	67.5 ± 19.0	-1.0 ± 22.3	64.4 ± 18.8	-8.6 ± 24.3	0.46	0.17
Cognitive function	78.6 ± 20.6	-0.4 ± 23.0	82.4 ± 20.6	-12.2 ± 23.9	0.31	0.06
Emotional well-being	57.1 ± 22.8	5.4 ± 43.2	59.5 ± 16.7	-21.6 ± 42.5	0.65	**0.014**
Role limitation- emotional	70.6 ± 43.3	0.4 ± 27.2	78.9 ± 36.3	-6.4 ± 21.0	0.37	0.22
Satisfation with sexual function	60.4 ± 26.7	2.0 ± 34.9	54.3 ± 30.6	-3.7 ± 37.0	0.33	0.40
Change in health	47.1 ± 19.9	0.5 ± 21.3	50.6 ± 22.7	4.6 ± 35.5	0.43	0.36

*p-values refer to t-test between treated and untreated groups at baseline and after 2 years follow-up.

**Relapse rate was evaluated in the 2-years preceding study entry (baseline)and after 2 years of follow-up (change)

Results of multivariate hierarchical analysis are summarized in Table 3, 4, 5. *At baseline *a poor physical health composite score was significantly associated with a higher age at inclusion (p = 0.043) and higher EDSS, FSS and BDI scores (p < 0.0003). A lower mental health composite score was correlated to increased BDI (p < 0.0001) and FSS score (p = 0.019). Other unfavourable factors were the male gender (p = 0.005) and RR course (p = 0.037). As expected, almost all QoL scales related to the physical composite score showed a negative association with EDSS and FSS score. All mental QoL scales were mainly related to impaired BDI (Table 4 and 5). Treatment did not influence physical and mental composite scores and relative subscales at baseline (Table 3, 4, 5).

**Table 3:** Predictors of MSQoL-54 composite scores by IFN-β treatment (Multivariate hierarchical analysis)

	**Physical Health Composite Score**		**Mental Health Composite Score**	
Effect	β	p	β	p
Intercept*	65.08	**<.0001**	69.31	**<.0001**
Age at inclusion	-0.19	**0.043**	-0.22	0.06
Gender (Male vs. Female)	-1.51	0.43	-6.95	**0.005**
Disease duration	-0.1	0.41	-0.04	0.82
Disease course (SP vs. RR)	3.69	0.24	8.39	**0.037**
Relapse rate 2-yrs before study entry	0.69	0.75	0.62	0.83
EDSS	-3.44	**0.0003**	-0.85	0.47
BDI	-0.59	**<.0001**	-1.44	**<.0001**
FSS	-6.69	**<.0001**	-2.08	**0.019**
MMSE-c	-0.28	0.44	0.45	0.34
*IFN-β treatment (Yes vs No)*	-0.09	0.97	2.75	0.36
Relapse rate change after 2-yrs	2.15	0.42	0.60	0.84
EDSS change after 2-yrs	-1.57	0.26	1.59	0.31
BDI change after 2-yrs	-0.60	**<.0001**	-1.08	**<.0001**
FSS change after 2-yrs	-5.21	**<.0001**	-2.48	**0.01**
MMSE-c change after 2-yrs	-0.35	0.53	-0.16	0.81
Time**	1.32	0.54	3.33	0.19
***Time × IFN-β treatment (Yes vs No)***	-1.65	0.58	-9.19	**0.01**

*Intercept value represents the baseline mean score for reference group (Untreated)

**Time value represents the mean score change for reference group (Untreated) after 2 years of follow-up

**Table 4:** Predictors of Physical health MSQoL-54 scales by IFN-β treatment (Multivariate hierarchical analysis)

	**Energy**		**Health perceptions**		**Physical function**		**Role limitation physical**		**Bodily pain**		**Social function**	
Effect	β	p	β	p	β	p	β	p	β	p	β	p
Intercept*	45.11	**<.0001**	44.67	**<.0001**	69.40	**<.0001**	71.72	**<.0001**	74.23	**<.0001**	72.54	**<.0001**
Age at inclusion	-0.12	0.36	0.06	0.68	-0.4	**0.01**	-0.60	**0.03**	-0.36	**0.04**	0.06	0.66
Gender (Male vs. Female)	-0.51	0.84	2.00	0.52	1.07	0.75	-17.3	**0.003**	3.74	0.31	-0.32	0.90
Disease duration	-0.02	0.92	-0.36	0.08	0.10	0.64	0.19	0.60	-0.38	0.12	-0.30	0.10
Disease course (SP vs. RR)	9.54	**0.03**	7.35	0.16	-8.44	0.12	8.77	0.34	17.33	**0.004**	8.65	0.06
Relapse rate 2-yrs before study entry	0.37	0.90	-2.01	0.58	0.70	0.86	1.39	0.83	-0.20	0.96	3.76	0.24
EDSS	-2.33	0.06	-3.54	**0.02**	-8.44	**<.0001**	-4.69	0.09	-2.82	0.11	-2.65	**0.05**
BDI	-1.16	**<.0001**	-0.26	0.20	0.25	0.26	-0.92	**0.01**	0.22	0.36	-0.66	**0.0004**
FSS	-4.80	**<.0001**	-6.8	**<.0001**	-8.69	**<.0001**	-10.0	**<.0001**	-9.74	**<.0001**	-6.72	**<.0001**
MMSE-c	-0.19	0.71	-0.43	0.46	0.05	0.94	-1.90	0.10	0.02	0.98	-0.30	0.57
***IFN-β treatment (Yes vs No)***	1.43	0.65	3.56	0.32	1.20	0.77	5.03	0.50	4.54	0.32	0.23	0.94
Relapse rate change after 2-yrs	4.96	0.17	2.40	0.61	3.76	0.20	-2.71	0.69	2.96	0.54	6.77	0.08
EDSS change after 2-yrs	-0.11	0.95	-1.49	0.54	-4.54	**0.003**	0.59	0.86	0.70	0.77	-2.72	0.16
BDI change after 2-yrs	-0.67	**0.0005**	-0.48	**0.05**	-0.14	0.37	-1.59	**<.0001**	-0.82	**0.002**	-0.67	**0.001**
FSS change after 2-yrs	-5.20	**<.0001**	-4.89	**0.001**	-4.65	**<.0001**	-4.75	0.03	-5.98	**0.0001**	-4.39	**0.0003**
MMSE-c change after 2-yrs	-1.09	0.15	0.20	0.84	-0.10	0.87	-3.04	0.04	-0.64	0.53	0.18	0.81
Time**	8.67	**0.004**	3.36	0.37	-4.84	**0.05**	-1.00	0.86	-1.81	0.65	0.64	0.84
***Time × IFN-β treatment (Yes vs No)***	-0.76	0.86	-1.72	0.74	-6.15	0.07	-2.54	0.77	3.82	0.50	-1.73	0.69

*Intercept value represents the baseline mean score for reference group (Untreated)

**Time value represents the mean score change for reference group (Untreated) after 2 years of follow-up

**Table 5:** Predictors of Mental health MSQoL-54 scales by IFN-β treatment (Multivariate hierarchical analysis)

	**Emotional well being**		**Role limitation emotional**		**Health distress**		**Cognitive function**		**Overall quality of life**	
Effect	β	p	β	p	β	p	β	p	β	p
Intercept*	73.6	**<.0001**	53.68	**<.0001**	69.6	**<.0001**	78.3	**<.0001**	68.6	**<.0001**
Age at inclusion	-0.31	0.32	-0.11	0.45	0.08	0.57	-0.41	**0.01**	0.02	0.86
Gender (Male vs. Female)	-19.83	**0.003**	1.68	0.57	-3.69	0.19	-8.55	**0.01**	-4.05	0.14
Disease duration	-0.29	0.49	0.13	0.50	0.08	0.69	-0.16	0.49	0.13	0.49
Disease course (SP vs. RR)	15.2	0.15	8.75	0.08	4.21	0.37	14.8	**0.008**	-2.55	0.57
Relapse rate 2-yrs before study entry	-0.31	0.97	0.15	0.96	-0.31	0.92	-0.40	0.92	-1.40	0.66
EDSS	1.15	0.71	-2.13	0.14	-0.11	0.93	-0.70	0.67	-1.51	0.25
BDI	-1.50	**<.0007**	-1.86	**<.0001**	-1.75	**<.0001**	-1.01	**<.0001**	-1.35	**<.0001**
FSS	-8.33	**<.0005**	1.67	0.12	-2.86	**0.005**	-1.06	0.38	0.02	0.98
MMSE-c	0.15	0.91	0.42	0.44	0.84	0.11	1.69	**0.009**	0.39	0.46
***IFN-β treatment (Yes vs No)***	7.67	0.34	5.22	0.13	4.99	0.13	3.12	0.44	-1.16	0.73
Relapse rate change after 2-yrs	-4.36	0.57	3.64	0.39	-1.90	0.72	2.90	0.51	-3.34	0.35
EDSS change after 2-yrs	2.95	0.44	-1.32	0.54	1.57	0.56	3.09	0.17	-0.24	0.89
BDI change after 2-yrs	-1.65	**<.0001**	-1.20	**<.0001**	-0.84	**0.003**	-0.91	**.0001**	-1.15	**<.0001**
FSS change after 2-yrs	-3.63	0.13	-1.14	0.38	-3.66	**0.02**	-0.20	0.88	-2.65	0.02
MMSE-c change after 2-yrs	-1.29	0.42	0.58	0.50	1.42	0.20	0.04	0.97	-1.40	0.6
Time**	5.68	0.37	5.31	0.11	4.15	0.32	-0.64	0.86	0.31	0.91
***Time × IFN-β treatment (Yes vs no)***	-19.11	**0.03**	-4.95	0.27	-12.29	**0.03**	-9.65	0.05	2.53	0.54

*Intercept value represents the baseline mean score for reference group (Untreated)

**Time value represents the mean score change for reference group (Untreated) after 2 years of follow-up

*At Follow-up*, worsening in physical and mental QoL health composite scores (Table 3) and in most of their subscales (Table 4 and 5) were significantly associated to higher BDI and FSS scores. Changes in EDSS did not influence all QoL subscales, except for physical function (p = 0.003). When considering the impact of the treatment on QoL after two years, the untreated patients did not show statistical changes in physical and mental health composite score. The IFN-β treated group showed a worsening in physical composite score and in most of their relative subscales, even if it was not significant. In this group mental health composite score significantly worsened over two years (p = 0.01). In addition emotional well being (p = 0.03) and health distress (p = 0.03) showed significant negative changes.

Thirty-four patients (28.8%) did not complete the QoL questionnaire at two years follow-up: 27 belonged to untreated and 7 to treated group. The assessment of informative drop-outs did not show significant differences in all the clinical and QoL measures at baseline between drop-outs and completers (data not shown).

## Discussion

To our knowledge, this study is the first to evaluate the influence of the therapy on QoL by multivariate hierarchical analysis and the first to demonstrate a poor QoL in relation to IFN-β treatment.

At inclusion time treated MS patients showed a younger age, a lower disease duration and a higher relapse rate in the two years before study entry in comparison to untreated group. This latter finding could be explained by the short period of the treatment at study entry, and in turn it represented the reason of starting the treatment. However, the used statistical analysis adjusted for all clinical and demographic inter-group differences. Previous short and long term IFN-β studies indicated a positive influence of the treatment on physical QoL [14,26,27] and mental QoL [28]. Moreover MS patients showed a good performance in mental QoL domains, also independently on the treatment (29, 30). In our study short-term treatment did not influence physical and mental QoL. In fact no differences in QoL scores were found between untreated and treated patients. After two years we observed a slight, even if not significant, worsening in physical QoL in treated patients, and this was in agreement with another report [31]. The main finding of our analysis was the negative influence of IFN-β treatment on mental health composite score and in most of its relative subscales QoL over the time.

In accordance with several authors fatigue and depression are undoubtedly significant predictors of a poor QoL, affecting both physical and mental domains, even after adjusting for all other clinical factors [6,8,10,11,32]. Although fatigue and depression may be considered the most frequent side effects during IFN therapy, we found that their impact on QoL was independent on the therapy, since FSS and BDI scores did not differ in treated and untreated patients. Furthermore changes in fatigue and depression over time induced a progressive worsening of QoL.

Clinical disability at inclusion was related to a poor physical QoL [1,6-8,11,30], but its negative influence was not as much as expected [29]. In fact changes in EDSS over the time did not impact significantly QoL measures. Disease duration did not affect the QoL, whereas a higher age at inclusion was significantly related to a poor physical composite score as well as to physical function, role limitation physical, bodily pain and cognitive function. Previous studies reported lower QoL score in SP in comparison to RR MS (6, 29, 32). In contrast, we found that RR course negatively influenced QoL. In our MS population the SP course was much less frequent than RR course and this might contribute to a bias in the analysis.

Certainly the relative short period of follow-up, in comparison to previous longer longitudinal studies, may represent a possible limitation of the present study. [30]. Further analysis in a larger number of treated patients is required in order to evaluate the influence of frequency and route of administration of IFN-βs

## Conclusion

In conclusion this study showed that treatment with IFN-β had a negative impact on QoL over the time in MS patients, influencing in particular the mental composite score. On the contrary, short-term treatment did not affect the QoL. Depression and fatigue were confirmed as main predictors of a poor QoL, whereas clinical disability had a minor unfavourable impact. In planning interventions for MS, psychological components of patients have to be always taken into account. Prospective studies in a larger sample of MS patients are required to enhance the evidence of the drug impact on QoL [33].

## Competing interests

All authors give no competing financial and non-financial interests to declare

## Authors' contributions

ILS conceived and coordinated the study and drafted the manuscript; AC participated in the study design and helped to draft the manuscript; CT participated in the study design and helped to draft the manuscript; AB carried out the clinical evaluation; FP carried out the statistical analysis; IP carried out the clinical evaluation; MFD carried out the neuropsychological evaluation; ML carried out the neuropsychological evaluation; FG carried out the clinical evaluation; PL contributed to design the study
